# Identifying a critical skeletal muscle threshold and systemic predictors for acute urinary retention in benign prostatic hyperplasia with sarcopenia: an internally validated model

**DOI:** 10.3389/fendo.2026.1965871

**Published:** 2026-09-23

**Authors:** Qizhi Yang, Yuan Xu, Shuang Hou, Bo Yang, Lin Huang

**Affiliations:** 1 Department of Urology, Affiliated Hospital of Guizhou Medical University, Guiyang, China; 2 Organ Transplantation Department, Affiliated Hospital of Guizhou Medical University, Guiyang, China

**Keywords:** acute urinary retention, benign prostatic hyperplasia, inverse probability of treatment weighting, restricted cubic spline, sarcopenia

## Abstract

**Background:**

The concurrent presentation of benign prostatic hyperplasia (BPH) and sarcopenia complicates the clinical evaluation of acute urinary retention (AUR). This study aimed to identify clinical predictors and explore non-linear associations with AUR risk in this specialized comorbidity.

**Methods:**

A retrospective cohort of 328 adult males with comorbid BPH and sarcopenia was analyzed. Stabilized inverse probability of treatment weighting (IPTW) was utilized to adjust for baseline confounding. Feature selection was conducted via Lasso penalization (lambda.1se threshold) to prevent overfitting. Non-linear relationships were evaluated using restricted cubic splines (RCS) with three fixed knots. The predictive model underwent internal validation via 1,000 bootstrap resamples.

**Results:**

Multivariable weighted logistic regression identified older age and catheterization history as potential risk factors for AUR, whereas higher skeletal muscle index (SMI) and pharmacological treatments (5-ARIs, alpha-blockers) were associated with reduced risk. RCS analysis indicated a significant non-linear relationship (P-non-linear = 0.002), suggesting a sharp increase in AUR probability when SMI declined below a critical threshold. The internally validated model exhibited an area under the curve of 0.789 and adequate calibration.

**Conclusions:**

Identifying specific muscle depletion thresholds and selective clinical indicators may assist in stratifying AUR risk among patients with BPH and sarcopenia.

## 1 Introduction

Benign prostatic hyperplasia (BPH) is a highly common condition in older men, often associated with lower urinary tract symptoms which may progress to acute urinary retention (AUR) (1, 2). AUR is an acute and bothersome urological event which may require emergency catheterization and surgery (3). In the modern era of geriatric medicine, patients with BPH seldom present with only urological disease, and they often have multiple age-related diseases, including sarcopenia, the loss of muscle mass over time (4). BPH and sarcopenia constitute a combined clinical profile in the elderly population; however, risk factors for the onset of AUR in the context of this comorbidity are not extensively studied (5, 6).

In addition to changes that occur at the local level in the prostate, the pathogenesis of clinical progression in older men is now considered in the context of systemic interactions (7). There is a potential pathophysiological link between inflammatory and metabolic alterations and muscle degradation (8). As a result, it has been suggested that systemic parameters such as the systemic immune-inflammation index (SII) and triglyceride-glucose (TyG) index may reflect the general inflammatory and metabolic status (9, 10). Conventional analytical methods, however, tend to use linear relationships when assessing these continuous indices of systemic and sarcopenic factors (11). These assumptions may not reflect possible non-linear threshold effects, especially in vulnerable groups where reserves are limited.

While several recent clinical studies and meta-analyses have established standard urological risk factors for AUR in BPH patients, the specific impacts driven by age-related muscle depletion and systemic environments remain less clear. There are also significant methodological difficulties in building predictive models for AUR in specific comorbid groups. Specific cohorts of patients with a combination of specific comorbidities are often small in size. Traditional propensity score matching can lead to the loss of substantial numbers of un-matched patients in these relatively small data sets, with a subsequent loss of statistical power (12). In addition, it may be difficult to properly penalize the use of multiple candidate predictors in standard multivariable logistic regression models, which may contribute to over-fitting and reduce the external validity and generalizability of the results (13, 14). Such strict statistical adjustments, specific for the small sample size, are therefore necessary to help ensure that predictive associations are reliable.

We sought to identify predictors of AUR in the specific comorbidity of BPH and sarcopenia by employing an inverse probability of treatment weighting (IPTW) strategy, which harmonizes baseline confounders while preserving the complete dataset (15, 16). To strictly comply with events-per-variable guidelines and prevent model overfitting, candidate features were filtered through a conservative Lasso penalization algorithm (17). Our analytical framework further incorporated restricted cubic splines with predefined knots to explore potential non-linear risk patterns, followed by rigorous bootstrap internal validation and decision curve analysis to evaluate the clinical applicability of the finalized model (18, 19).

## 2 Materials and methods

### 2.1 Study design and ethical considerations

Approval for all study protocols was granted by the Medical Ethics Committee of China Guizhou Aviation Industry Group 302 Hospital (Approval Number: KJKYLL-2025-11). Because this research was an observational analysis relying entirely on de-identified electronic medical records from a retrospective cohort, the institutional review board exempted the investigators from obtaining written informed consent. Throughout the entire data collection and analytical process, all procedures remained in full compliance with the ethical frameworks established by the 1975 Declaration of Helsinki and its later amendments.

### 2.2 Study population

Sarcopenic patients were adult males with a concurrent diagnosis of benign prostatic hyperplasia (BPH). The inclusion criteria were as follows: (1) clinical diagnosis of BPH with documented LUTS, (2) concurrent presence of sarcopenia, which was assessed using the SMI (appendicular skeletal muscle mass divided by height squared) measured via bioelectrical impedance analysis (BIA), adhering to the Asian Working Group for Sarcopenia (AWGS) 2019 consensus guidelines (defined as an SMI < 7.0 kg/m ² for males), and (3) availability of complete baseline clinical and laboratory information.

Patients were excluded from the study if they (1) had a history or diagnosis of prostate cancer or other urological malignancies at presentation; (2) were diagnosed with a neurogenic bladder, urethral stricture, or active UTI at presentation; (3) had severe hepatic or renal insufficiency; (4) were found to have a history of active inflammatory or hematological diseases or immunosuppressive therapy; or (5) had incomplete medical records regarding key study variables or clinical outcomes. After applying these criteria, 328 patients were finally enrolled for analysis.

### 2.3 Data collection and variable definitions

Baseline demographic and clinical data were extracted from the institutional electronic medical record system. The collected variables included age, body mass index (BMI), prostate volume (PV), International Prostate Symptom Score (IPSS), SMI, triglyceride-glucose (TyG) index, serum albumin (ALB), and the systemic immune-inflammation index (SII). Additionally, pharmacological interventions prior to the clinical event, specifically the usage of 5-alpha reductase inhibitors (5-ARI) and alpha-blockers, alongside any history of catheterization, were recorded. The primary outcome of this study was the occurrence of acute urinary retention (AUR) (including both first-episode and recurrent events requiring acute intervention) documented at the time of the current hospital admission or during the corresponding inpatient clinical observation period.

### 2.4 Statistical analysis

Propensity scores were used to create stabilized inverse probability of treatment weighting (IPTW) to minimize confounding at baseline. An absolute standardized mean difference (SMD) of <0.10 was used to assess the covariate balance.

A Lasso-penalized logistic regression was used for feature selection and the IPTW matrix was included. The conservative 1-standard-error threshold calculated using 10-fold cross validation (1se value) was used. Selected variables were then added to a multivariable weighted logistic regression to calculate adjusted odds ratios (ORs) and 95% confidence intervals (CIs).

In the restricted cubic splines (RCS), three fixed knots were used in the weighted cohort to explore possible non-linear relationships, and P-values for overall and non-linear associations were computed. Generalizability was tested by performing subgroup analyses with multiplicative interaction terms. 1,000 bootstrap resamples were used for internal validation with computation of the optimism-corrected area under the curve (AUC), calibration curves, and Brier scores. Finally, clinical usefulness was assessed by decision curve analysis (DCA) and individual predictions were graphically displayed as a waterfall plot. Analysis was carried out using the R software, and a two-sided P value < 0.05 was deemed statistically significant. The development and reporting of the predictive model were conducted in accordance with the Transparent Reporting of a multivariable prediction model for Individual Prognosis or Diagnosis (TRIPOD) guidelines (**Table 1**).

## 3 Results

### 3.1 Baseline characteristics and evaluation of IPTW adjustment

A total of 328 patients with combined BPH and sarcopenia were identified and were divided into two groups: Low SII group (n = 164) and High SII group (n = 164). Before weighting, there were some significant baseline differences in clinical variables as summarized in **Table 1** (Age, SMI, and Serum Albumin; all P < 0.05). For example, the distribution of SMI before IPTW was statistically different between the two groups (P<0.001) (**Figure 1E**).

**Figure 1:**
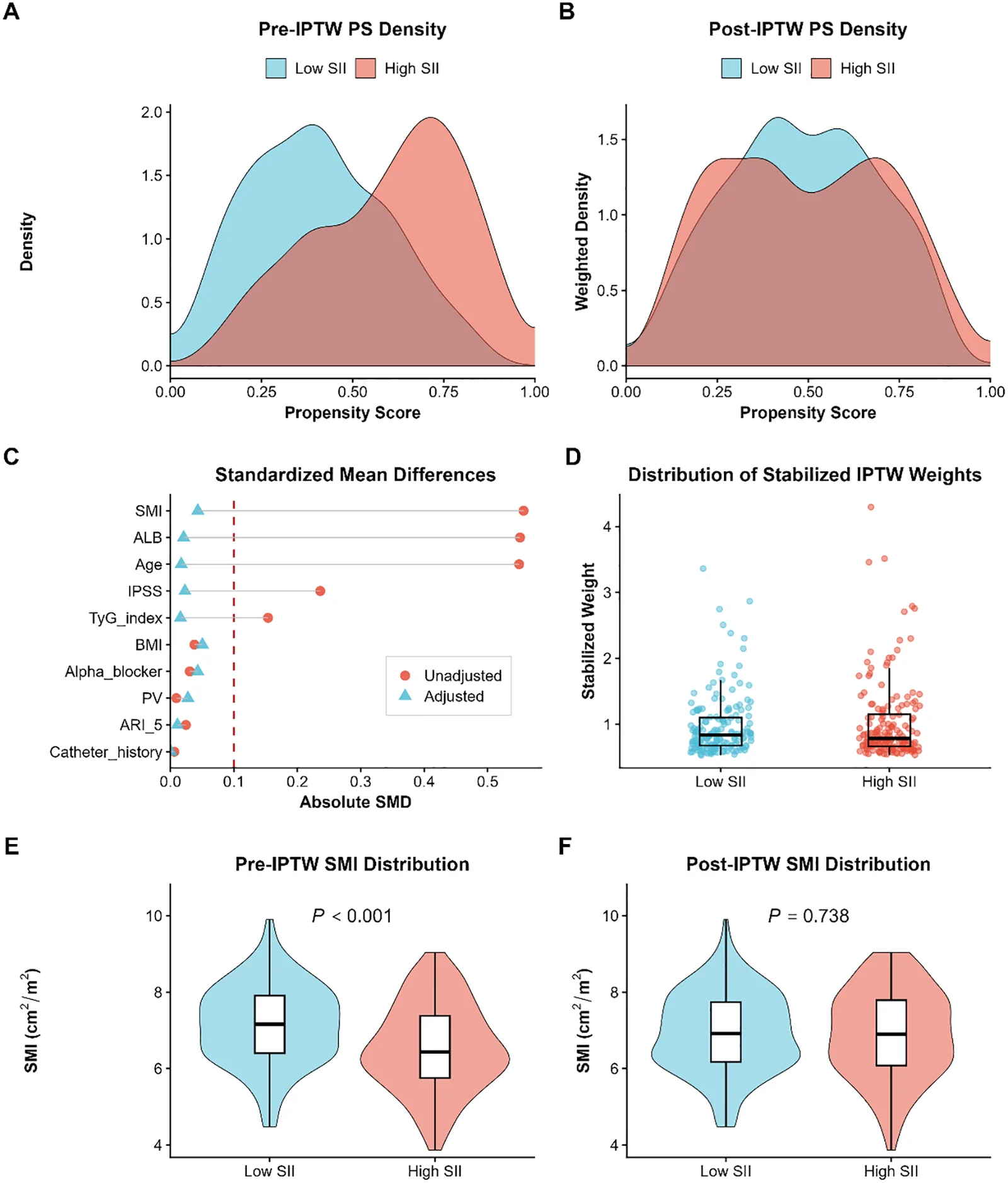
Propensity score distribution and assessment of covariate balance before and after stabilized inverse probability of treatment weighting (IPTW). **(A, B)** Propensity score density plots before and after IPTW showing the overlap between groups. **(C)** Baseline covariate absolute standardized mean differences (SMDs) plotted on a Love plot. The vertical dashed line is the SMD threshold of 0.1. **(D)** Distribution of the stabilized weights used in the cohort. **(E, F)** Violin and box plots of the distribution of the skeletal muscle index (SMI) for the low and high SII groups before and after weighting. The P-values are based on unweighted and weighted t-tests, respectively.

Stabilized inverse probability of treatment weighting (IPTW) was used to adjust for these baseline confounders without compromising the sample size. The density plots of propensity scores showed that the overlap between the two groups increased after the weighting (**Figures 1A, B**). Moreover, the range of distribution of the assigned stabilized weights was limited and reasonable (**Figure 1D**). This method was used to avoid potential scale over-dominance and model instability associated with extreme values of weight.

Baseline characteristics were well balanced as per Post-IPTW assessment. All covariates included were absolute standardized mean differences (SMDs) of < 0.10 (**Figure 1C**; **Table 1**). In fact, the preobserved difference in SMI was neutralized in the weighted cohort (P = 0.738, **Figure 1F**), which suggests that the weighted cohort was comparable within the measured clinical variables.

**Table 1:** Baseline characteristics of the bph-sarcopenia cohort before and after inverse probability of treatment weighting (IPTW).

Characteristic	Unweighted Low SII (N=164)	Unweighted High SII (N=164)	Unweighted P-value	Unweighted SMD	IPTW Weighted Low SII (N=159.4)	IPTW Weighted High SII (N=167.1)	Weighted P-value	Weighted SMD
Age, years	70.4 (5.1)	73.5 (6.1)	< 0.001	0.55	71.7 (5.5)	71.8 (5.9)	0.894	0.017
BMI, kg/m2	24.0 (3.3)	24.1 (2.8)	0.733	0.038	24.0 (3.2)	24.2 (2.9)	0.682	0.05
Prostate Volume, mL	70.0 (58.5-84.4)	71.5 (56.4-89.3)	0.766	0.009	70.2 (59.5-84.6)	74.2 (56.9-89.3)	0.82	0.028
IPSS, points	20.0 (16.0-23.0)	20.5 (17.0-24.0)	0.05	0.236	20.0 (16.0-23.0)	19.0 (17.0-24.0)	0.863	0.023
Skeletal Muscle Index, cm2/m2	7.1 (1.0)	6.5 (1.1)	< 0.001	0.557	6.9 (1.0)	6.9 (1.2)	0.738	0.043
TyG Index	8.7 (0.6)	8.8 (0.6)	0.165	0.154	8.8 (0.6)	8.8 (0.5)	0.894	0.016
Serum Albumin, g/L	39.3 (3.4)	37.6 (2.9)	< 0.001	0.551	38.5 (3.4)	38.4 (3.0)	0.874	0.021
Alpha-blocker Usage, n (%)	129 (78.7%)	124 (75.6%)	0.599	0.03	124.8 (78.3%)	123.7 (74.0%)	0.432	0.043
5-ARI Usage, n (%)	75 (45.7%)	71 (43.3%)	0.739	0.024	70.1 (44.0%)	71.7 (42.9%)	0.858	0.011
Catheter History, n (%)	27 (16.5%)	28 (17.1%)	0.999	0.006	27.1 (17.0%)	28.4 (17.0%)	0.987	0.001
Acute Urinary Retention (AUR), n (%)	39 (23.8%)	88 (53.7%)	< 0.001	N/A	50.4 (31.6%)	79.4 (47.5%)	0.013	N/A

Data are presented as Mean (SD) for normally distributed continuous variables, Median (IQR) for non-normally distributed continuous variables, and count (percentage) for categorical variables. After Inverse Probability of Treatment Weighting (IPTW), absolute Standardized Mean Differences (SMDs) for all baseline covariates were less than 0.10, indicating excellent covariate balance. Acute Urinary Retention (AUR) was the primary outcome and therefore not included in the weighting algorithm (SMD = N/A). Independent sample t-tests, Wilcoxon rank-sum tests, and Chi-square tests were used for unweighted comparisons; weighted generalized linear models (survey-weighted t-tests and Chi-square tests) were used for IPTW-adjusted comparisons.

Interestingly, the primary outcome, acute urinary retention (AUR), was not included in the weighting algorithm and was therefore not included in the SMD (**Table 1**). However, the incidence of AUR was still statistically higher in the High SII group than in the Low SII group in the IPTW adjusted cohort (47.5 percent vs. 31.6 percent, P = 0.013). This observation indicates a possible association between high systemic immune inflammation status and AUR risk in this population and can be investigated in a multivariable fashion.

### 3.2 Feature selection and multivariable weighted logistic regression

However, to determine the strongest possible predictors of acute urinary retention (AUR) without the risk of model overfitting in a relatively small sample, a penalized Lasso regression model was created in the IPTW-weighted data set. The shrinkage of the candidate variables as the tuning parameter (lambda) increases is shown in the Lasso coefficient profile plot (**Figure 2A**). The 10-fold cross validation method was used to find the optimal value of lambda. The lambda.1se is a model that is designed to be more parsimonious and to follow the events-per-variable (EPV) statistical guidance. Rather than using the lambda.min (**Figure 2B**), the 1se threshold was chosen.

**Figure 2:**
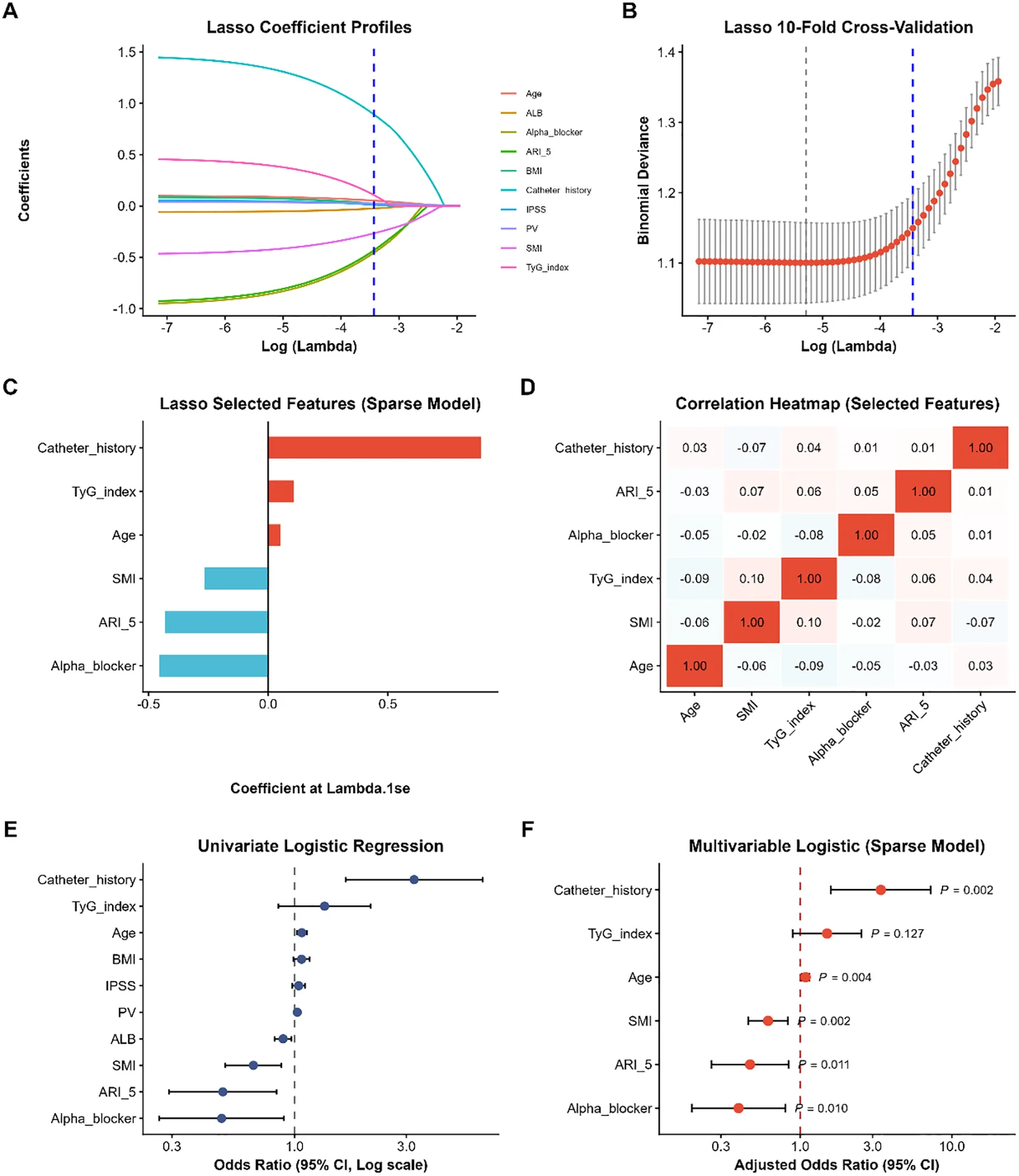
Feature selection using Lasso regression and weighted logistic regression analyses for acute urinary retention (AUR) risk. **(A)** Lasso coefficient profile plot of the candidate variables. **(B)** Ten-fold cross-validation curve for tuning parameter (lambda) selection. The right dashed line indicates the lambda.1se value used to establish a sparse model. **(C)** Bar chart presenting the non-zero coefficients for the features selected by the Lasso model. **(D)** Spearman correlation heatmap among the selected features. **(E)** Forest plot of univariate weighted logistic regression for AUR risk. **(F)** Forest plot of multivariable weighted logistic regression based on the Lasso-selected features. Odds ratios (ORs) and 95% confidence intervals (CIs) are presented on a logarithmic scale.

With this conservative criterion, the original candidate pool was successfully reduced to a sparse model with only six key features with non-zero coefficients: Catheter history, TyG index, Age, SMI, 5-ARI usage and Alpha-blocker usage (**Figure 2C**). A subsequent Spearman correlation analysis between these selected features showed that all the correlation coefficients between two features were under 0.15 (**Figure 2D**), which indicated that there was no significant multicollinearity between these features, so they could be used in the subsequent multivariable analysis.

After identifying the features, univariate and multivariable logistic regression analysis was performed in the weighted cohort. Based on the results of the univariate analysis, there were several variables that were potentially associated with AUR risk (**Figure 2E**). Five of the six variables included in the multivariable model were still statistically significant (**Figure 2F**) after the six features were selected by Lasso. In particular, older Age and history of catheterization were suggested as risk factors for AUR (both P < 0.01). In contrast, an increased SMI, and use of 5-ARIs and alpha-blockers, seemed to be correlated with a lower risk of AUR (all P < 0.05). The Lasso algorithm maintained TyG index to maintain the metabolic aspect of the model, but multivariable TyG index was not independently associated with AUR (P = 0.127). The results provide an indication that SMI and certain pharmacological treatments may be important in the clinical profile of AUR in this comorbid population.

### 3.3 Non-linear association analysis using restricted cubic splines

To further explore any potential non-linear associations between various important continuous variables and the risk of AUR, we then examined the association between each of these variables and AUR in an exploratory fashion. To do this, restricted cubic spline (RCS) models were used in the IPTW adjusted cohort. For all RCS models, the number of knots was set conservatively to 3 in order to avoid the risk of overfitting the model in the presence of a small sample size and to smooth spurious fluctuations at the distributional tails.

The RCS analyses showed various associations between the clinical indices. Interestingly, there was a notable non-linear relationship between the Skeletal Muscle Index (SMI) and risk of AUR (P-overall < 0.001, P-non-linear = 0.002; **Figure 3C**). The risk trajectory for AUR was relatively stable when the SMI was in the higher range, but a notable threshold was observed, with a rapid escalation of AUR risk as the SMI dropped below around 6.5. This non-linear association indicates that a threshold decrease of skeletal muscle mass may be closely related to the onset of AUR in this patient population.

**Figure 3:**
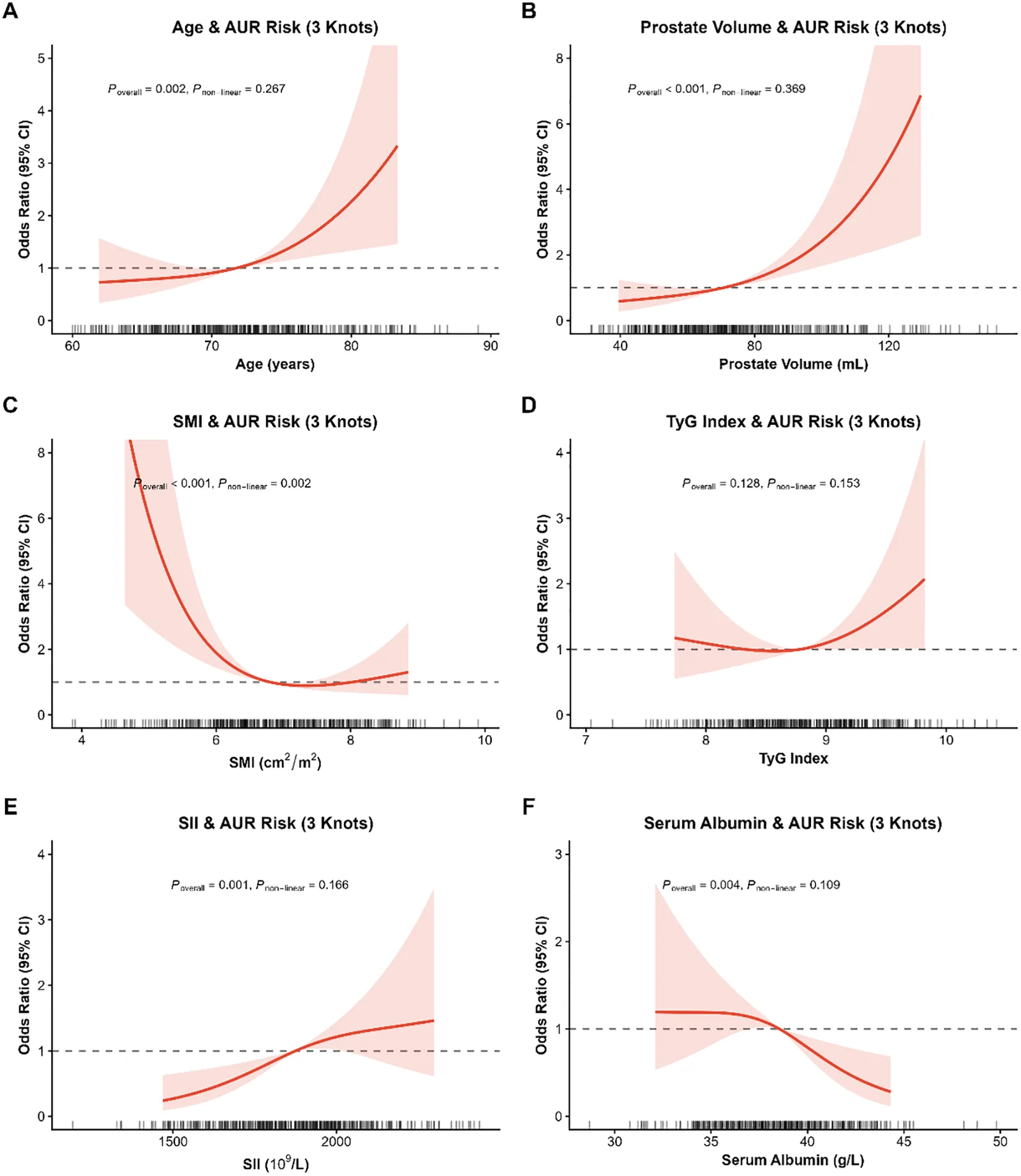
Restricted cubic spline (RCS) plots of the relationship between continuous variables and the risk of acute urinary retention (AUR). Three knots are fixed and the models are adjusted for selected covariates. The shaded areas are 95% confidence intervals, and the solid red lines are estimated odds ratios (ORs). The horizontal dashed line is the reference line, with an OR of 1.0. The data distribution is plotted as a rug plot at the bottom. **(A)** Age. **(B)** Prostate volume. **(C)** Skeletal muscle index (SMI). **(D)** TyG index. **(E)** Systemic immune-inflammation index (SII). **(F)** Serum albumin. The overall association is indicated by the P-overall value in each panel, and the P-non-linear value is provided to show whether there are non-linear trends.

Several other continuous variables showed mainly linear relationships to the risk for AUR. For example, there were significant overall associations to AUR for both Age (P-overall<0.01) and Prostate Volume (P-overall<0.01); however, their non-linear parts were not statistically significant (P-non-linear = 0.267 and 0.369 for Age and Prostate Volume respectively, **Figures 3A, B**) indicating a continuous and progressive risk profile. Likewise, there were significant linear trends for the Systemic Immune-Inflammation Index (SII) (P-overall = 0.001) and Serum Albumin (P-overall = 0.004) and no significant non-linearity (**Figures 3E, F**). Besides, there was no significant relationship between the TyG index and AUR in spline analysis overall or for non-linear effects (P-overall = 0.128, P-non-linear = 0.153; **Figure 3D**); the results were consistent with those of the above-mentioned Multivariable logistic regression. These differential dose-response profiles indicate the different clinical implications of the metabolic and sarcopenic indices when assessing AUR susceptibility.

### 3.4 Subgroup interactions, internal validation, and clinical utility

Subgroup analyses with multiplicative interaction terms were conducted to further test the generalizability of the predictive model to a variety of clinical scenarios. Association patterns were stable across the different demographic and disease severity strata, namely Age, Prostate Volume (PV) and Body Mass Index (BMI) as shown in **Figure 4A**. Importantly, P-interaction was not significant for any of these subgroups (all P-int > 0.50), suggesting that the model performance was not unduly altered by these patient baseline characteristics. Likewise, there were no significant multiplicative interactions (all P-int > 0.40; **Figure 4B**) among the treatment intervention subgroups (history of catheterization, use of alpha-blockers, and 5-ARI administration). These results indicate the generalizability of the model is stable in different clinical sub-cohorts.

**Figure 4:**
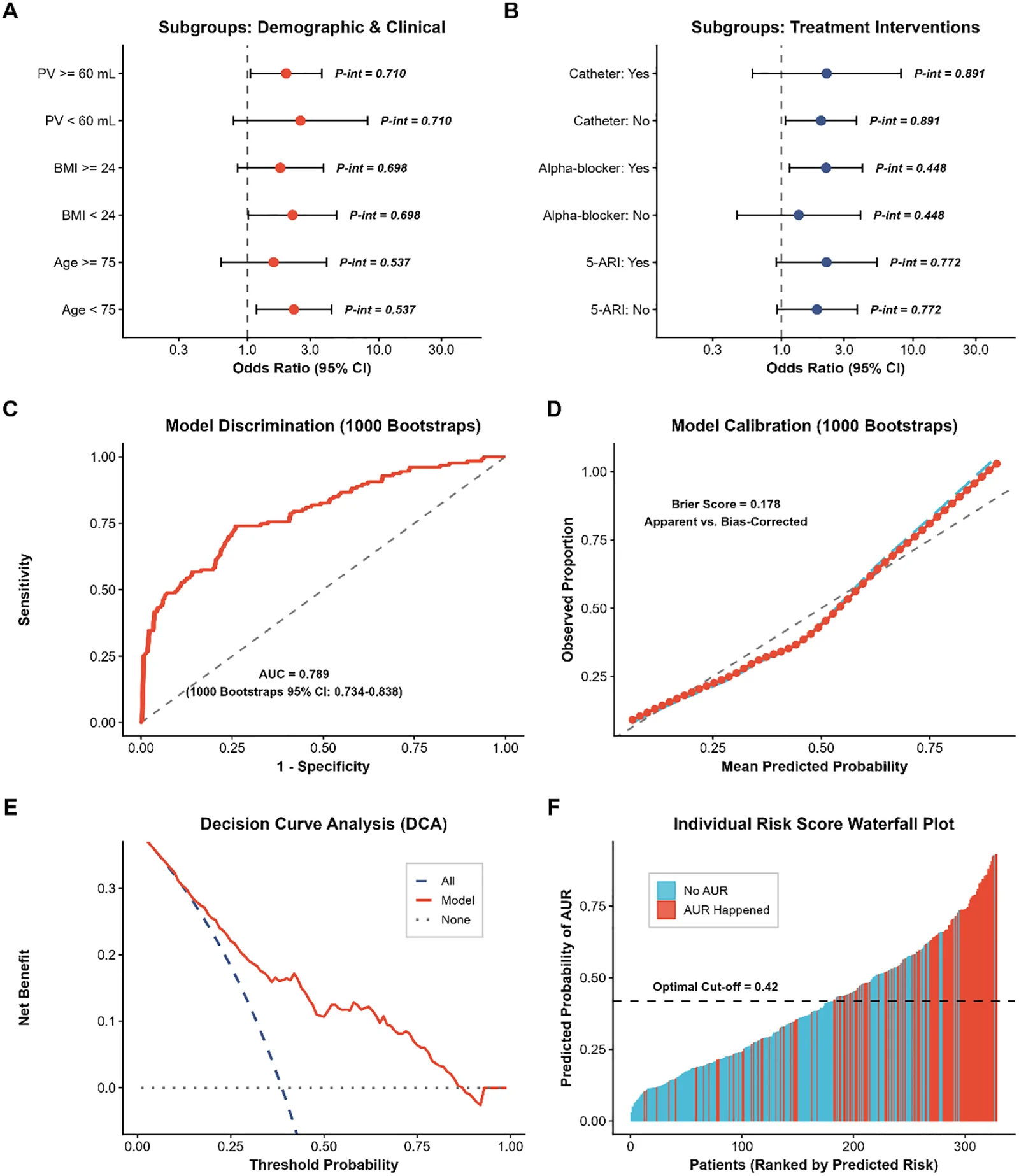
Subgroup analyses, internal validation, and individual risk visualization of the predictive model. **(A, B)** Forest plots showing subgroup analyses stratified by demographic/clinical characteristics and treatment interventions. P-interaction (P-int) values are provided to indicate potential multiplicative interactions. **(C)** Receiver operating characteristic (ROC) curve, with the area under the curve (AUC) calculated using 1000 bootstrap resamples. **(D)** Calibration curve of the model with 1000 bootstrap resamples. The diagonal dashed line represents ideal calibration, while the solid lines represent apparent and bias-corrected model performances. **(E)** Decision curve analysis (DCA) suggesting the net benefit of the predictive model across various threshold probabilities compared to treat-all and treat-none strategies. **(F)** Waterfall plot displaying the distribution of individual predicted probabilities for AUR. The horizontal dashed line represents the optimal cut-off value calculated by the Youden index, with colors indicating the actual observed clinical outcomes.

A stringent internal validation method (1000 bootstrap resamples) was used to assess the discriminatory power and calibration of the model, and to discourage overfitting, given the relatively small total sample size. The receiver operating characteristic (ROC) analysis revealed an area under the curve (AUC) of 0.789 with a 95 percent bootstrap confidence interval of 0.734–0.838 (**Figure 4C**) which showed acceptable discriminative ability. At the same time, the calibration curve showed a good match-up between the apparent predictions and the bias-corrected trajectory, with good agreement between the predicted probabilities and the observed proportion of AURs (Brier Score = 0.178; **Figure 4D**).

A decision curve analysis (DCA) was used to evaluate the potential practical use of the model. In a wide range of threshold probabilities (0.05 to 0.85), the DCA showed that use of the predictive model might provide a greater net clinical benefit than would the hypothetical “treat-all” and “treat-none” strategies (**Figure 4E**). Finally, an individual risk score waterfall plot was created to help illustrate the range of predicted probabilities for the cohort. Using the best cut-off point of 0.42 as determined by the Youden index, the plot showed that actual AUR events clustered around the patients with a predicted probability > 0.42 (**Figure 4F**). The visualization herein indicates that the model might be useful in a clinical environment to stratify high risk individuals.

## 4 Discussion

Benign prostatic hyperplasia (BPH) and sarcopenia are common conditions in the geriatric population, but the ability to predict acute urinary retention (AUR) in the setting of the two conditions has not been well studied. Using inverse probability of treatment weighting (IPTW) and Lasso-penalized regression, we found that low skeletal muscle index (SMI) was a significant predictor of AUR risk, as was a number of different systemic metabolic and inflammatory parameters, both in linear and non-linear fashion, as well as certain pharmacological histories (20, 21). From a scientific reasoning perspective, the relationships observed between these systemic indices and clinical outcomes might reflect a complex, interconnected environment. Specifically, elevated systemic inflammation (assessed via indices like SII) and insulin resistance (proxied by TyG) might be associated with prostatic edema, altered autonomic sympathetic tone, or subtle microvascular changes in the lower urinary tract. Furthermore, systemic metabolic dysfunction is frequently coupled with muscular degradation. Consequently, sarcopenia might reflect a broader physiological decline that compromises pelvic floor integrity or detrusor reserve, thereby potentially diminishing the physical capacity required to overcome bladder outlet obstruction.

One interesting finding in our analysis was that there was a non-linear relationship between SMI and AUR risk. The muscle depletion has been considered as a continuous linear variable in the epidemiological studies in geriatric urology, but the restricted cubic spline analysis in our study indicated a threshold effect (22, 23). The splines were carefully designed without any more knots than necessary given the sample size, to avoid excessive tail fluctuations and overfitting. In this conservative setting, there did not seem to be a gradual increase in risk of AUR, but rather a sharp increase in risk when SMI fell below a certain threshold. This statistical trend suggests that a risk increment may not be linear, but may need to focus on critical loss levels when assessing muscle mass in relation to AUR (24, 25).

Among the limitations of the current study is the methodological design that was adjusted for the issues of confounding and dimensionality among such a relatively small cohort. Since the traditional propensity score matching approach generally is associated with a reduction in the number of subjects in each group, IPTW was used to ensure the balance of the baseline differences but to keep the entire cohort of 328 patients (26). In addition, to comply with the statistical guideline of events-per-variable, the choice of features was regulated by the conservative lambda. In Lasso cross-validation, the value of 1se was calculated. Surprisingly, penalization algorithm retained TyG index, but TyG index was not statistically significant according to conventional criteria in the multivariable logistic model. This retention indicates that the metabolic dimensions are also associated with the overall predictive profile but perhaps not as strongly when included with the sarcopenic and pharmacological variables. Moreover, the model’s retention of older age and catheterization history might suggest that advanced chronological age and prior lower urinary tract instrumentation represent surrogate markers for prolonged disease duration or baseline functional vulnerability. Conversely, the use of alpha-blockers and 5-ARIs appeared to be associated with a reduced probability of AUR, consistent with their established therapeutic roles in alleviating smooth muscle tension and prostatic volume. Importantly, our findings suggest that sarcopenia might indeed indicate an increased risk of AUR in BPH patients, particularly as muscle mass (SMI) drops below a critical physiological threshold, underscoring the potential relevance of evaluating systemic muscular health alongside local prostatic parameters. Additionally, while catheterization history emerged as a strong predictor, it is important to note that this variable primarily encompassed temporary catheterizations for unrelated perioperative care or mobility restrictions, rather than intractable recurrent AUR. Therefore, its predictive value might reflect an underlying baseline vulnerability of the lower urinary tract rather than a novel pathophysiological pathway.

Subgroup and validation analyses were also performed to assess the generalizability and potential clinical application of the results. The fact that there are no substantial multiplicative interactions between different demographic and clinical strata suggests the predictive associations are fairly consistent across different profiles. To perform internal validation, 1000 bootstrap resamples were used, due to the limitations of the sample size. This method allowed for the penalized estimation of the discriminative ability, and calibration without the additional loss of statistical power due to splitting the data. Also, the waterfall plot of the individual risk score provided a practical visual overview of the traditional nomograms, which could help identify in the clinical setting those who might be at high risk (1, 27).

Despite the rigorous statistical adjustments, this study has several main limitations. First, since the study was retrospective and single center, the external validity of the results might be limited, and although IPTW was performed to balance observed covariates, unmeasured residual confounding cannot be ruled out. Specifically, detailed urological parameters such as intravesical prostatic protrusion (IPP), post-void residual (PVR) volume, specific severity scores of LUTS, underlying bladder stones, or potential underactive bladder were not fully available in our database. The omission of these established confounders might introduce bias into the analytical modeling and current conclusions. Importantly, well-established predictors of BPH progression, such as prostate-specific antigen (PSA), post-void residual (PVR) urine volume, and peak urinary flow rate (Qmax), were not uniformly available in our retrospective dataset and were consequently omitted from the models, which might introduce bias into the comprehensive risk evaluation. Second, the analysis was based on baseline measurements taken at a single point in time, and thus does not allow assessment of how dynamic (longitudinal) changes in skeletal muscle mass or systemic inflammatory indices would be associated with subsequent AUR events.

## 5 Conclusion

Using stabilization of inverse probability of treatment weighting (IPTW) in these patients with comorbid benign prostatic hyperplasia and sarcopenia, multivariable models revealed specific clinical and pharmacological risk factors for AUR. Restricted cubic spline analysis indicated that mainly the skeletal muscle index exhibited a non-linear relationship with AUR with a prominent threshold effect, while systemic inflammatory and metabolic parameters mostly showed linear trends. The internally validated predictive model, optimized by conservative Lasso penalization, showed fairly good discriminative ability and calibration but without apparent overfitting. This is further supported by incorporation of individual risk score visualizations and decision curve analysis, suggesting the potential practical applicability of this approach. These results indicate that the inclusion of certain sarcopenic thresholds and the use of selective clinical indicators may help to stratify the risk of AUR; however, future prospective external validations are needed to test its validity on a larger scale.

## Data Availability

The raw data supporting the conclusions of this article will be made available by the authors, without undue reservation.
